# Medication Adherence in Mental Health Care: Examining Gender Differences in an Omani Sample

**DOI:** 10.1192/bjo.2026.11198

**Published:** 2026-06-30

**Authors:** Mohammed Qutishat, Salim Al-Huseini, Mandhar Almaqbali, Mohammed Albalushi

**Affiliations:** 1 College of Nursing, Sultan Qaboos University, Muscat, Oman; 2 Al Masarra Hospital, Ministry of Health, Muscat, Oman; 3 Ministry of Health, Muscat, Oman; 4 Al Masarra Hospital, Muscat, Oman

## Abstract

**Aims:**

Mental health issues in Arab countries, including Oman, have been traditionally stigmatized, leading to increased non-adherence to prescribed medications. This study aims to explore gender differences in medication adherence among patients with mental illness in Oman, highlighting the impact of social factors on treatment compliance.

**Methods:**

A cross-sectional study was conducted at Al Masarra Hospital in Muscat between October 2020 and May 2021. Using convenience sampling, 302 participants aged 18 and older, receiving follow-up care for documented mental health disorders, were recruited. The Medication Adherence Rating Scale (MARS) was employed to assess adherence levels and collect demographic data.

**Results:**

The study included 302 participants, predominantly aged 26–45 years (59.61%). Male patients had a mean medication adherence score of 5.75 (SD=2.550), compared to 5.67 (SD=2.347) for females. An independent samples t-test revealed a statistically significant difference, with a t-value of−2.027 (p=0.044), suggesting that gender influenced adherence rates. ANCOVA showed that marital status (F(1, 295)=5.321, p=0.021) and patient insight (F(1, 295)=7.456, p=0.007) significantly predicted adherence, whereas no significant effect of gender was observed.

**Conclusion:**

This study highlights the complexity of medication adherence in mental health care, emphasizing the need for tailored interventions that consider gender dynamics and social support systems. By addressing these factors, mental health services can improve adherence rates and overall patient outcomes in Oman.

